# Schooling progress, learning reversal: Indonesia’s learning profiles between 2000 and 2014

**DOI:** 10.1016/j.ijedudev.2021.102436

**Published:** 2021-09

**Authors:** Amanda Beatty, Emilie Berkhout, Luhur Bima, Menno Pradhan, Daniel Suryadarma

**Affiliations:** a Mathematica, 955 Massachusetts Avenue, Cambridge, MA, 02139, USA; b AIGHD, Amsterdam Institute for Global Health and Development, Paasheuvelweg 25, 1105 BP, Amsterdam, the Netherlands; c The SMERU Research Institute, Jl. Cikini Raya No.10A, RT.13/RW.5, Cikini, Jakarta, 10330, Indonesia

**Keywords:** International education, Development, Educational policy, Curriculum, Learning profiles, Indonesia

## Abstract

We examine the relationship between schooling completed and mathematics learning from 2000 to 2014 by developing learning profiles for Indonesia. Using nearly-nationally representative survey data, we find a large gap between students’ ability and standards set by the national curriculum. Learning declined over 14 years, a loss of a fourth of a standard deviation. To put this loss in context, the average child in grade 7 in 2014 achieved the same numeracy mastery as the average child in grade 4 in 2000. The reduction in learning was widespread, affecting all subgroups. Junior and senior secondary enrollment increased over this timeframe, but this decline was not due to changes in student composition.

## Highlights

• We use nationally representative data to create mathematics learning profiles for Indonesia.
• We compare student learning levels and changes in learning from 2000 to 2014 to curriculum expectations. Students’ mastery of basic skills is low.
• Over 14 years, learning declined by approximately 0.25 standard deviations.
• The average child in grade 7 in 2014 had learned as much as the average child in grade 4 in 2000.
• Changes in learning were not driven by changes in student composition.

## 1 Introduction

Over the past twenty years, Indonesia has made dramatic progress in improving junior and senior secondary enrollment. While the country had achieved universal primary enrollment in 1988 (Government of Indonesia, 1998), between 2000 and 2014, the timeframe of this study, Indonesia saw a 17 percentage point improvement in junior secondary enrollment, to 77 percent, and a 20 percentage point improvement in senior secondary enrollment, to 59 percent (Statistics Indonesia, 2020).

Simultaneous with extending years of schooling for millions of children, the country also made massive investments in education with the stated goal of improving quality. In 2002, the 1945 Constitution was amended to require that 20 percent of the budget be allocated to education spending. In 2005, the government passed the Teachers and Lecturers Law, which required higher qualification standards for new and existing teachers and effectively doubled civil servant teacher salaries (UU No. 14, 2005). Indonesia’s move to decentralization in 2001 also extended to education policy such that its approximately 500 districts could make decisions on education delivery and adjust policy to local context and needs (UU No. 22, 1999).

Despite reforms that provided more educational resources, raised standards, and increased school access, the country continues to face learning challenges. In 2018 Indonesia scored 379 out of 500 on the mathematics portion of the Programme for International Student Assessment (PISA); a score of 379 is 7th from the lowest score among the nearly 80 countries or states taking the test (OECD, 2019). PISA defines Level 2 as “achieving at least a minimum proficiency level,” and the Sustainable Development Goals (SDG) use PISA “Level 2” as a metric for SDG Target 4.1 (UNESCO, 2018). Fewer than 1 in 3 students in Indonesia were able to perform at Level 2 or above in mathematics (OECD, 2019). Indonesia demonstrated similar results in the Trends in International Mathematics and Science Study (TIMSS) in 2015, in which 27 percent of 4th graders did not even met the lowest benchmark defined as having “some basic mathematical knowledge.” Another 50 percent met the lowest benchmark, 23 percent met benchmarks 2 or 3, and no students met the highest benchmark (Mullis et al., 2016). Looking at Indonesia’s historic performance on these assessments in mathematics, it has largely stayed the same over time for PISA (OECD, 2019) and fallen for TIMSS since 2003 (Mullis et al., 2004; Mullis et al., 2008; Mullis et al., 2012).

This article takes a deeper look at the contrast between the positive trends in enrollment and the more negative or static international assessment findings on learning. It could be that newer learners entering the system (i.e., possibly students from households with less educational exposure, facing greater challenges staying in school, or keeping up with the instructional pace) bring down average learning. It could also be that learning at least did not go up because the system’s quality deteriorated; or the answer could be a combination of these explanations. We explore this contrast using a unique longitudinal household-level dataset, the Indonesian Family Life Survey (IFLS). The IFLS includes variables on household characteristics and mathematics assessments for children age 7 and up in 2000 and 2014. We use the testing data to develop mathematics learning profiles that show learning by age and grade-level; and we assess how learning varies by background characteristics and over time. We are able to examine the trends in learning for in-school and out-of-school children, in contrast to international assessments, which only assess in-school children. Moreover, we can assess learning changes with rising enrollment in Indonesia.

To better understand how learning changed in the face of this improvement in enrollment, we first answer the following questions, using the ILFS 2014 for children across all schooling-relevant ages: What did children in school know compared to curriculum expectations? How much did in-school children learn as they progressed through school? These two questions allow us to frame children’s basic numeracy competencies within the context of what the education system expects children to know by a particular grade and examine if schooling is delivered more learning with each additional year. Then we ask: Did learning change over time? Specifically, we compare learning profiles of all children and of enrolled children between 2000 and 2014. This is one of two studies that analyses learning accumulation in Indonesia across different years. Afkar et al. (2018) looked at mathematics learning for in-school children between 2011 and 2012; we utilize data for all school-age children from 2000, 2007, and 2014.

We finally answer the question: Did different subgroups demonstrate different learning profiles? We pursue this analysis in order to understand if one group is driving our findings and examine if different groups disproportionally benefited from or were disadvantaged by education system changes during this timeframe. We look at separate effects for children in different wealth groups, males and females, children whose mothers have different education levels, and different provinces.

### 1.1 Changes to Indonesia’s educational landscape between 2000 and 2014

In this section we offer context to our research questions regarding whether, for whom, and why learning may have changed from 2000 to 2014. We describe changes to the education landscape during that timeframe, including the shift towards decentralization, rising enrollment, increased education spending, lower teacher-student ratios, improved teacher qualifications, curriculum changes that focus less time on mathematics, and eliminating class grades as a criterion for graduation.

Indonesia generally, and its education system specifically, went through dramatic changes starting in 1999 when the country transitioned to democracy, which included a shift towards decentralization, offering more financial and political autonomy to its now 514 districts. In 2003, the government solidified this initiative in education by granting more autonomy to districts to manage education (UU No. 20, 2003). Since 2003, civil servant teachers have been hired by the central Ministry of Education and Culture (MoEC), which also sets the curriculum, upper-grade assessments, and accredits schools; but districts distribute and manage teachers, hire and fire non-civil servant teachers, allocate funding to schools, manage school infrastructure, and carry out a range of other functions. This move towards decentralization meant that the country saw more geographic variation in education delivery than it had in previous decades.

**Fig. 1.1:**
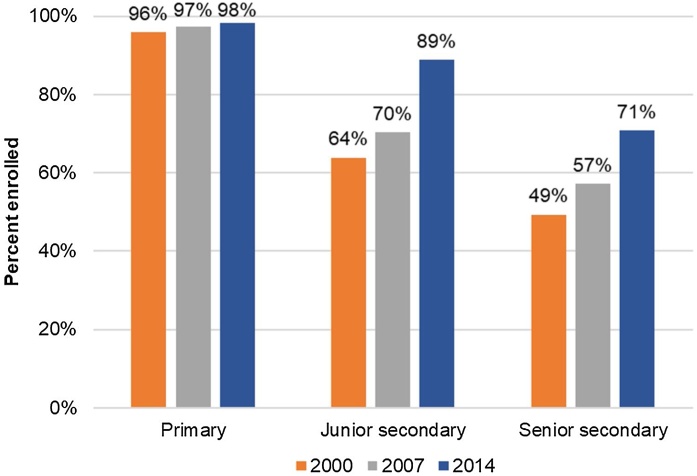
Educational enrollment by year and school level. **Note**: The figure shows the total of net enrollment and completion rates. Net enrollment and completion rates are calculated as a percentage of respondents who are within the anticipated age range and who (1) ever enrolled in the specified school level and are still enrolled, or (2) ever enrolled in the specified school level and finished that school level: 7- to 12-year-olds for primary school, 13- to 15-year-olds for junior secondary school, and 16- to 18-year-olds for senior secondary school.

**Source**: IFLS 3, 2000), IFLS 4, 2007), and IFLS 5, 2014

Enrollment had already begun to rise at the primary level (grades 1–6) before 1999 as primary school attendance had been compulsory since 1984 (UU No. 20, 2003), and primary enrollment was near universal since 1988 (Government of Indonesia, 1998). Junior secondary (grades 7–9) schooling, which became compulsory in 2003, and senior secondary (grades 10–12) schooling saw significant enrollment growth during our study period, 2000−2014. The IFLS 2014 data show that junior secondary enrollment increased by 19 percentage points, from 71 percent to 90 percent; and senior secondary enrollment increased by 24 percentage points, rising from 47 percent in 2000 to 71 percent in 2014 (Fig. 1.1).^1^ (The IFLS dataset is described in detail in Section 1.3) These figures were 79 percent for junior secondary and 61 percent for senior secondary nationally in 2019 (Statistics Indonesia, 2020).^2^

Not surprisingly, attainment for people ages 20–30 also reflect these enrollment trends. Between 1993 and 2014, average years of schooling increased from 7.1 years to 10.5 years (authors’ analysis of IFLS). In 2014, according to the IFLS, 95 percent had completed primary school; this attainment went up slightly between 2000 and 2014, from 91 percent. In 2014, this figure was 82 percent for junior secondary and 57 percent for senior secondary, up from 64 percent and 38 percent respectively in 2000. There was also little within-school-level drop-out among 20–30-year olds. Almost 95 percent of students who enrolled at any level of schooling completed it.

Government spending on education grew significantly over our study period. In 2002, the government amended the 1945 Constitution to require that 20 percent of the budget be allocated to education spending. Indonesia achieved this goal in 2009, nearly doubling spending on education over just five years (World Bank, 2013). By 2014, spending per year reached over 300 trillion Rupiah or nearly US$21 billion (World Bank, 2018a). A large share of the increased funding for education was spent on employing more teachers and driving down class sizes. The student-teacher ratio was 22−1 in 1999; and even in the midst of increasing enrollment was 16−1 by 2010, one of the lowest ratios in the region (UNESCO, 2018). A larger education budget was also spent on increasing pay for teachers as stipulated in the 2005 Teachers and Lecturers Law, although research demonstrated that this did not affect learning (de Ree et al., 2018).

Teachers became on average more highly educated over this timeframe. Between 2003 and 2016, due to changes to teacher certification requirements resulting from the 2005 Teachers and Lecturers Law, the share of teachers with a bachelor’s degree rose from 37 to 90 percent (World Bank, 2018a). There is evidence that teachers’ education may not explain much variation in teacher effectiveness in developed countries (Hanushek et al., 2005); in Indonesia, teachers with bachelor’s degrees performed slightly better on a series of math, science, and Indonesian test questions than teachers with less education (de Ree, 2016).

While we might not expect spending or improved teacher qualifications to improve learning, we would not expect those improvements to have a negative effect. We now discuss several changes – children’s exposure to mathematics content and national examination incentives – that could have negatively affected learning over the study period. Curriculum changes reduced the number of hours of math instruction per week. The 1994 curriculum mandated 10 hours a week of math instruction for grades 1–3 and eight hours a week for grades 4–6. In 2004, the curriculum required teachers in grades 1–3 to teach math “thematically,” which meant that teachers were to cover all academic subjects related to a theme or topic; and lowered math instruction limits to five hours per week for grades 4–6 (Sugiarti, 2014). Shifting to thematic lessons was an adjustment for teachers who received little training or guidance in implementing this approach. The curriculum change could have prompted teachers to cover less material, but it is also possible that teachers found it challenging to teach with less structured guidance.

The 2003 National Education System Law changed the significance of leaving exams. Prior to 2003, a student’s graduation from 6th, 9th or 12th grade was based on yearly grades and national exam results. After 2003, the country took a lower stakes approach of basing promotion on a combination of teacher discretion and the leaving exams. Districts also took over responsibility for the grade 6 leaving exam, so the content varied by district, although MoEC’s testing center still had responsibility for overseeing the junior secondary and senior secondary leaving exams. In 2014, grade 6 and 9 exam scores still had stakes in some areas as they could have been used for admission to junior secondary and senior secondary schools, and admission to some schools was highly competitive.

### 1.2 Learning profiles literature

A learning profile is a plot of skills, knowledge, or subject-matter competence across multiple grades or ages, among in-school and/or out-of-school children. It represents the skill or knowledge that a cohort of children accumulates during schooling (Kaffenberger, 2019). Kaffenberger (2019) identifies three main categories of learning profiles: contemporaneous cross-section (knowledge across a cross-section of respondents in different grades and ages), adult retrospective (knowledge of a cross-section of adults who have completed schooling), and true panel (knowledge of the same respondents over time). This study uses IFLS to generate contemporaneous cross-section and true panel profiles.

The majority of studies that employ learning profiles use contemporaneous cross-section. Assessments by organizations such as the ASER (Annual Status of Education Report) Centre, Uwezo, and USAID, which created the EGRA/MA (Early Grade Reading Assessment and Early Grade Math Assessment), generated some of the first examples of learning profiles in developing countries. For example, Jones et al. (2014) used Uwezo data to show that in Kenya, Tanzania, and Uganda more than half of 10-year-olds and one-third of 13-year-olds could not recognize a single written word or recognize numbers. Spaull and Kotze (2015) showed that the poor-wealthy gap in Grade 3 was three grade levels. Pritchett and Beatty (2015) used ASER data to illustrate the concept of learning profiles and incongruence between curriculum pace and actual student learning.

Less common are adult retrospective and panel profiles. Kaffenberger and Pritchett (2020) created adult retrospective learning profiles across ten countries using Financial Inclusion Insight data with young adults ages 18 to 37, as did Pritchett and Sandefur (2020) who used DHS literacy data from women aged 25–34 in 51 countries. The longitudinal study, Young Lives, utilizes similar questions across four countries – Ethiopia, India, Peru, and Vietnam – and has in several papers demonstrated vast differences in learning gains over time across countries using panel learning profiles (Rolleston, 2014; Rolleston and James, 2015; Singh, 2020). Also using panel profiles, the LEAPS program in Punjab, Pakistan followed the same children over four rounds or years of schooling, highlighting learning changes as children transitioned from public to private school and vice versa (Andrabi et al., 2008; Bau et al., 2021).

For Indonesia, Afkar et al. (2018) produced the first study of learning profiles and the first panel profiles. They examined changes in math learning for 40,000 children in 360 primary and junior secondary schools over two sequential years (2011 and 2012), using anchor items that were similar across grades. They found that approximately 40 percent of students did not master basic numeracy questions after three years in school and that in many schools, learning did not keep up with curriculum expectations.

While profiles naturally differ across countries, a common theme across the papers cited above and others is that profiles are shallow in many low- and middle-income countries, meaning students learn little as they progress through school. This finding is consistent with the “learning crisis” message from the 2018 World Bank World Development Report. Afkar et al. (2018) illustrate how shallow the learning profile is in Indonesia. They find that the same number of students who can recognize numbers by the end of grade 2 can do one-digit multiplication by the end of grade 3, indicating that only those who can recognize numbers are the ones who will learn one-digit multiplication, i.e., those who are behind do not catch up.

Another common finding across the papers cited above is that in countries with shallow learning profiles, much of the potential gains in learning are through improvements in the quality of learning per grade rather than the expansion in schooling. For example, Singh (2020) uses panel profiles to make comparisons of different countries with differential schooling productivity and shows that the effect of another grade of schooling in Vietnam is 0.25 to 0.40 standard deviations higher than in other countries. Exposing students to a more productive schooling environment like that in Vietnam closes nearly all of the cross-country achievement gap for students in Peru and India and 60 percent of the students in Ethiopia. Similarly, in a context in which even the advantaged have shallow learning profiles, Akmal and Pritchett (2021) generate simulations using ASER and Uwezo data to show that even helping poor students achieve the attainment profiles of the rich doesn’t necessarily generate large learning gains. In India, Pakistan, and Uganda, just 60 percent of poor students would be numerate and able to read a simple story if they achieved the attainment levels of the rich.

### 1.3 Data

We construct learning profiles using three waves of the IFLS, collected in 2000 (IFLS 3), 2007 (IFLS 4), and 2014 (IFLS 5) (Frankenberg et al., 1995; Strauss et al., 2004, 2009; Strauss, Witoelar, et al., 2016). The IFLS is a panel survey, started in 1993, that follows the same households and their offspring (if household members form a new household) at each survey round. The over 30,000 respondents live in 13 of 27 provinces, and the survey is representative of 83 percent of the Indonesian population. The IFLS randomly selected enumeration areas (EAs) in each province from a nationally representative sampling frame used in the 1993 SUSENAS, a socioeconomic survey designed by the Indonesian Central Bureau of Statistics.^3^ Within each EA, households were randomly selected from the 1993 SUSENAS listings (Frankenberg et al., 1995). The 2000 and 2014 waves serve as the primary source for analysis presented in this paper; we also use the 2007 data for panel analysis in Section 2.2.

While the IFLS was primarily designed to measure demographic changes, it includes a multiple-choice numeracy test with nine items shown in Table 1.1. Different age groups took one of two versions of the test with different levels of difficulty. Test 1 is the first four items and Test 2 is the latter four items in Table 1.1. The one overlapping question (56/84) is shaded in grey and was included in both versions. All items are multiple choice with four answer options, except for the first three questions, which had three answer options. Table 1.1 shows which respondent groups took which test items in which years. For the analysis presented in this paper, we mainly use results from respondents between ages 7–18 because the analysis primarily focuses on school-age children.

The mathematics test was first included in the IFLS in 2000. Children aged 7–14 took Test 1 while 15 to 18-year-old adolescents took Test 2. In the 2007 and 2014 IFLS, adolescents 15 years old or above were asked to take Test 1 again if they also took it seven years earlier when they were between 7 and 14 years old. Therefore, of the respondents 15 years old and above, a large percentage took all ten items across the two versions in the same IFLS year (88 percent in 2007 and 71 percent in 2014). (These students took the overlapping item twice, so we characterize this as ten items total.) Table 1.1 also shows our mapping of the items to the skill or concept that a child should have mastered by a certain grade according to the 2006 and 2013 national curriculum standards (Badan Standar Nasional Pendidikan, 2006; Kementerian Pendidikan dan Kebudayaan, 2013).

**Table 1.1:** IFLS’s numeracy questions, expected grade mastery according to the curriculum, and ages in which children were tested in which IFLS year.

Numeracy skill	Test question	Expected grade level mastery	Ages tested 2000	Ages tested 2007	Ages tested 2014
2-digit subtraction	49-23	1	All 7-14	All 7-14 88% of 15-18	All 7-14 71% of 15-18
3-digit addition and subtraction	267+112-189	2			
1-digit addition and multiplication	(8+9)*3	3			
Subtracting fractions	1/3-1/6	4			
2-digit division	56/84	4	All 7-14 All 15-18	All 7-14 All 15-18	All 7-14 All 15-18
Order of operations	(412+213)/(243-118)	3	All 15-18	All 15-18	All 15-18
Decimals	0.76-0.4-0.23	4			
Calculating interest (Percent 1)	Ali put 75,000 rupiah in his savings account. If he receives 5% interest a year, how much interest does Ali receive on his savings after one year?	5			
Calculating percent (Percent 2)	If 65 % of people smoke, and the current population is 160 million, how many people do not smoke?	5			

**Notes**: Data source is IFLS 3, 2000, IFLS 4, 2007, and IFLS 5, 2014, and Badan Standar Nasional Pendidikan, 2006 and Kementerian Pendidikan dan Kebudayaan, 2013. We examined the 2006 and 2013 curricula to determine the grade in which the numeracy skill was covered in the curriculum; and to examine if there were changes due to curricula reforms. In the IFLS data, Test 1 is referred to as EK 1 while Test 2 is referred to as EK 2.

Table 1.2 shows the sample size for the numeracy test in each survey wave. We excluded from the analysis those individuals for whom the complete numeracy test is missing because they refused, could not be contacted, did not have enough time, or any other reason unrelated to competencies (5.5 percent of the sample). We also excluded those individuals for whom educational attainment is missing (0.1 percent of the sample for whom we have a numeracy score).

**Table 1.2:** Numeracy question sample sizes, ages 7–18.

	2000	2007	2014
Respondents interviewed (attempted + did not attempt numeracy test)	9579	9517	11,362
Respondents who answered at least one numeracy question	9208	9162	10,697
Percent of respondents who answered at least one numeracy question for whom we imputed at least one item*	21.5	16.7	14.7

**Note**: Table includes in- and out-of-school children. In our analysis we also include students above 18 years old who are still enrolled in senior secondary school. This amounts to 84 students in 2000, 80 in 2007 and 63 in 2014. These individuals are excluded from the table as they are over 18.

* Imputation methods discussed in Section 1.4.

### 1.4 Methods

As discussed above in Section 1.3, there are two versions of the numeracy test—an easy version (Test 1) and a more difficult version (Test 2). We applied a test equating procedure using Item Response Theory (IRT) to generate a measure of numeracy skills that is comparable between the two versions of the test and adjusts for question difficulty. To link the test versions, we employed a horizontal test equating procedure using the group of respondents that answered both versions, called anchor respondents.

Responses from the anchor respondents generated the difficulty level and discrimination power of each of the ten items.^4^ As mentioned above, there is one overlapping item in Test 1 and Test 2: 56/84. While the question is the same in both versions, the notation was slightly different ($\frac{56}{84}$). We chose to treat the overlapping question as separate questions in each version because one-third of the respondents who answered both versions gave two different answers.

To estimate each respondent’s numeracy score using IRT, we use a three-parameter logistic model (Eq. (1)). Three parameters, item discrimination power, item difficulty, and a guessing parameter, are used to determine the fourth parameter, which is student ability. The difficulty parameter relates to the ability of an individual, such that if the difficulty parameter is equal to the ability parameter, the individual is equally likely to answer correctly or incorrectly. The discrimination parameter reflects how fast the probability of success changes with ability near the item difficulty. The higher the discrimination parameter, the better the item can differentiate high ability students with those with low ability. Putting these parameters in a formula, the probability of person *j* providing a positive answer to item *i* is given by

(1) $$\text{Pr}\left(Y_{ij}=1,\theta_{j}\right)=c_{i}+(1-c_{i})\frac{\text{e}\text{x}\text{p}\{\alpha_{i}\left(\theta_{j}-b_{i}\right)\}}{1+\text{e}\text{x}\text{p}\{\alpha_{i}\left(\theta_{j}-b_{i}\right)\}}\;\theta_{j}∼N(0,1)$$

where *α_i_* represents the discrimination of item *i*, *b_i_* represents the difficulty of item *i*, *c_i_* represents the guessing correction called the *pseudo guessing parameter* and *θ_j_* is the latent trait (or ability) of person *j* (StataCorp, 2017). We present the results for θ and weigh them using sampling weights. We present Bayesian Markov chain Monte Carlo estimates of the latent ability θ.^5^

The ability parameter reflects the respondent’s numeracy skill level. Even though the limited number and scope of the items pose constraints to our numeracy skill measure, tests of psychometric properties of the measure show that the test items are adequate for the numeracy comparisons we make.^6^ We standardize the numeracy skill measure using the mean and standard deviation of grade 1 students in the 2000 sample and rescale the measure to have a mean of 0 and a standard deviation of 100 for grade 1 students in 2000. This way, our measure shows the improvement in learning relative to grade 1 in terms of grade 1 standard deviations.^7^ Throughout the paper, we call this the “standardized numeracy score.”

The numeracy test responses contain missing values, and we find that missing data patterns are systematic. We find that the share of missing values generally increases as the question difficulty increases, measured by the grade in which the items are expected to be mastered according to the curriculum, and that the highest share of missing values is concentrated among the youngest respondents (see Table A2.1). This provides evidence that the missing value patterns are associated with lower skills, so we infer that respondents likely left these questions blank because they didn’t know the answer. Because leaving these values out of our analysis would bias the results, we impute the missing items as if the respondent gave an incorrect answer. Table 1.2 shows the percent of observations that we imputed with an incorrect answer. We impute at least one item response on the test for 22 percent of the 2000 sample and 15 percent of the 2014 sample. As a robustness check, we also perform our analysis without imputed values and by imputing missing values with random guessing and find that the learning profiles are steeper when imputing with wrong answers, because ignoring missing values or imputing with random guessing inflates scores of children in lower grades who had the most missing values. However, it does not alter our conclusions about differences in learning between subgroups and learning over time (see Appendix 2).

**Fig. 2.1:**
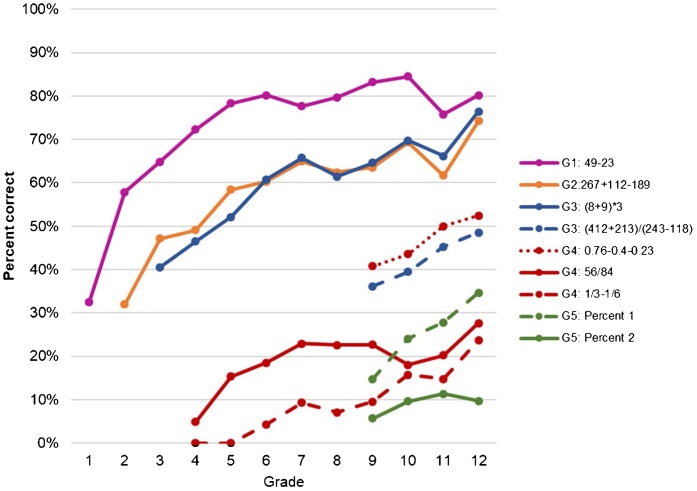
Learning by grade level and item, enrolled students in 2014. **Notes**: Results show the percent who answered each question correct among currently enrolled students. The sample sizes for each grade change depending on the number of children in that grade and what questions students should have mastered according to the curriculum per Table 1.1. Some results are presented beginning with students who enrolled in 9th grade as harder item-level questions were only asked among an older age group (15 years and older). Grade-level 1, 2 and one level 3 ((8+9)*3) questions have three answers; all remaining questions have four answers. The questions for Percent 1 and Percent 2 are in Table 1.1. Results are adjusted for guessing as described in Section 1.4.

**Source**: IFLS 5, 2014

For individual items shown in Fig. 2.1, we correct the percent correct for guessing such that, in expectation, a zero is given for those who randomly guessed and a 1 is given for those who knew the correct answer. As the test items are multiple choice, respondents could correctly answer a question by chance alone. To adjust for this guessing we use the following method (Eq. (2)) by Afkar et al. (2018). If α is the fraction that knows the answer and y is the fraction that answered correctly, then:

(2) $$y=\left(1-\alpha \right)\times \frac{1}{K}+\alpha \times 1$$

for K answer options. Those who guess have a probability of 1/K to answer correctly, while those who know the answers have a probability of one. We present the results for α and weight them using sampling weights.

In Section 2, we show the standardized numeracy score by gender, region (province), mother’s education level and wealth quintile. For the differences by wealth, we generate an asset index using Principal Component Analysis (PCA) at the household level (Filmer and Pritchett, 2001).^8^ For differences by region, we show the average difference in learning between 2000 and 2014 for the 13 provinces included in the IFLS.^9^ The IFLS data is representative at the provincial level (Frankenberg et al., 1995). We estimate the following regression model (Eq. (3)) using Ordinary Least Squares to measure the change in the standardized numeracy score between 2000 and 2014 within each of the provinces

(3) $$Y_{ipwg}=\beta_{1}+\beta_{2}W_{ipg}+\sum_{p=1}^{13}\beta_{2,p}.P_{iwg,p}+\sum_{p=1}^{13}\beta_{3,p}.W_{ip,g}.P_{iwg,p}+\gamma_{g}+\varepsilon_{ipwg}$$

Where Y is the standardized numeracy score for student *i* from province *p* in IFLS wave *w* in grade *g*. W is a dummy variable for the 2014 IFLS wave, P are dummy variables for the 13 provinces, γ_g_ are grade fixed effects, and ε is an error term.

## 2 Learning outcomes results

In this section we shed light on mathematics learning gains across grades in 2014 and from 2000 to 2014, using questions from the IFLS that were asked of respondents in both 2000 and 2014.

### 2.1 What did children in school know in 2014 compared to curriculum expectations? How much did in-school children learn from one grade to the next?

Our first finding is that learning levels were low in 2014 and by extension, children did not keep up with curriculum expectations. Fig. 2.1 shows descriptive learning profiles for the 2014 IFLS questions for each grade, by item, indicating what grade level the item content is covered in the curriculum. Just 67 percent of students in grade 3 could answer the simplest grade 1 question, 49−23, correctly. This low level of learning is even more pronounced for more “difficult” questions, such as those requiring calculating fractions or percent. Only 36 percent of 12th graders could correctly answer a word problem on calculating interest (Percent 1 in Fig. 2.1) and no 5th graders could answer 1/3−1/6, a grade 4 question, correctly.

Second, children learned little as they progressed through school. There was particularly little improvement in most numeracy skills after primary school (grade 6). For example, using the grade 1-level question, 49−23, which just 65 percent of grade 3 students could answer, we find that this mastery improved by approximately 15 percentage points by 6th grade, but there was no improvement between grades 7 and 12. The solid-line grade 1–3 items shown in Fig. 2.1 start with around 30–40 percent of students correctly answering the problem in the relevant grade level. In subsequent grades in primary school, the share of students correctly answering the question grew by approximately just 5–10 percentage points per grade; this share fell to 1 percentage point per grade in junior secondary school. For the items only asked of students in grades 9–12, the share of students answering correctly generally only improved by 1–4 percentage points per grade, with the exception of the percent problem regarding interest (Percent 1 in Fig. 2.1) for which we see up to a 5 percentage point improvement per grade in the share of students answering correctly in grades 9–12.

**Fig. 2.2:**
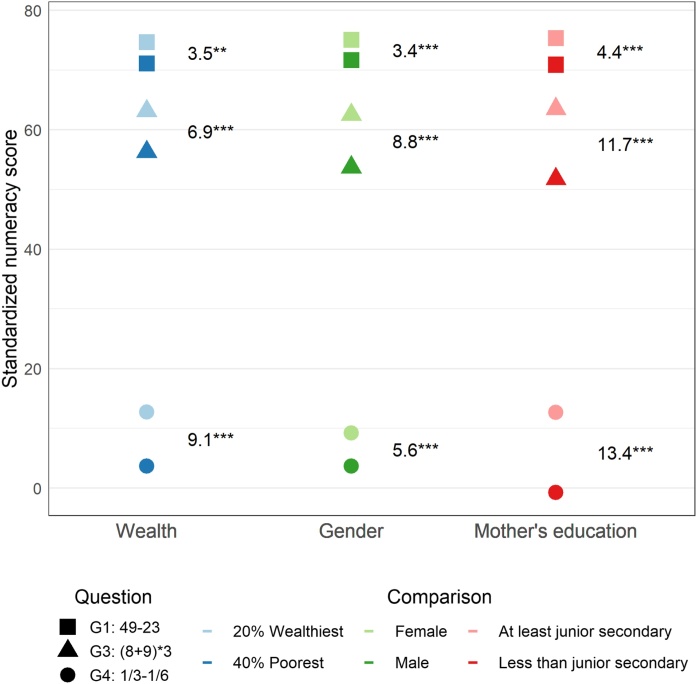
Subgroup differences for three questions, enrolled students in 2014. **Notes**: Results show the subgroup standardized numeracy score of the three different items and the subgroup difference among currently enrolled (40 percent poorest, males, and students with mothers who completed less than junior secondary school). The sample sizes for question change depending on the number of children enrolled in grades in which students should have mastered the question according to the curriculum per Table 1.1. For example, the students included in bars for the G4 question are enrolled in grade 4–12. Results are adjusted for guessing as described in Section 1.4. * p-value < 0.1 ** p-value < 0.05 *** p-value < 0.01

**Source**: IFLS 5, 2014

Looking at subgroup differences for these items, we find that differences grow with question difficulty, as shown in Fig. 2.2. While there was hardly any difference (3 percentage points) between the wealthiest 20 percent and the poorest 40 percent of the population in the grade 1 level question (49−23), this difference was 9 percentage points with a grade 4 level question (1/3−1/6). We find the largest difference between students whose mothers completed at least junior secondary school and students whose mothers completed less than junior secondary school. Students with mothers with higher attainment were 13 percentage points more likely to correctly answer the grade 4 question, while almost none of the students whose mothers completed less than junior secondary school could answer that question. For the hardest question, the smallest subgroup gap is that between males and females, yet there is still a 5 percentage point difference. All differences are statistically significant.

**Fig. 2.3:**
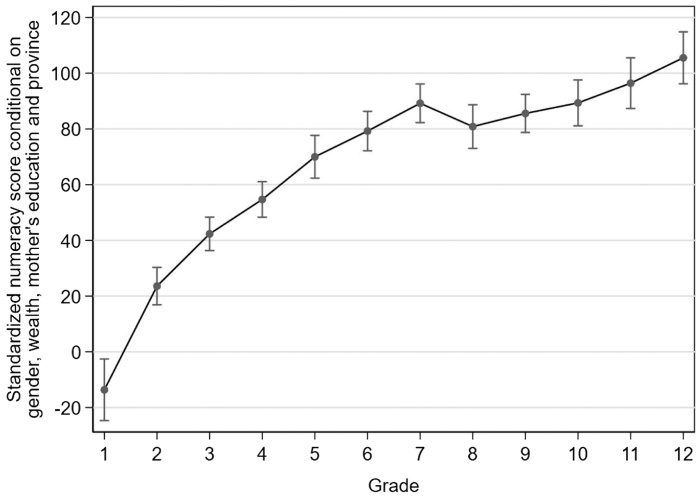
Change in standardized numeracy score due to an additional year of schooling controlling for gender, mother’s education, wealth quintile, and province.

**Source**: IFLS 5, 2014 **Notes**: Point estimates and 95 percent confidence interval for progress in numeracy score relative to grade 1. Standard errors are corrected for clustering at the enumeration are level and observations are weighted using survey weights. The controls are gender, a dummy indicating if the child’s mother’s completed at least junior secondary school, wealth quintile (poorest 40 percent, middle 40 percent or wealthiest 20 percent) and province dummies. Results are adjusted for guessing as described in Section 1.4.

**Fig. 2.4:**
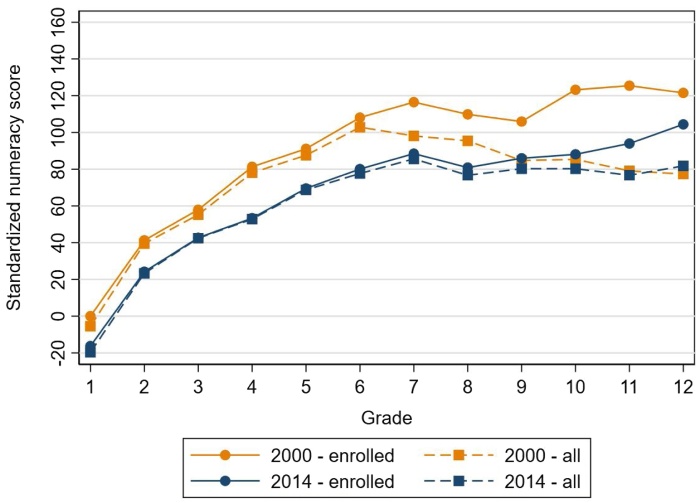
Standardized numeracy score in 2000 and 2014 by grade level completed (for enrolled children) or grade level they would have completed (for all enrolled and unenrolled children). **Note**: Results are adjusted for guessing as described in Section 1.4.

**Source**: IFLS 3, 2000 and IFLS 5, 2014

In addition to looking at performance on each individual question by current grade level, we use IRT to develop a numeracy score that incorporates responses to all questions and adjusts for question difficulty, as discussed in Section 1.4. Recall that we normalize the scores to have a mean of 0 and a standard deviation of 100 for grade 1 students in the year 2000 to get to the standardized numeracy score. Fig. 2.3 shows the score gains from an additional year of schooling from grades 2–12, relative to grade 1, using data from 2014. We control for gender, whether the child’s mother completed junior secondary school, wealth quintile, and province. The controls do not alter these results much (see Fig. 2.4, in Section 2.2, for the 2014 learning profile without controls), so differences in student composition across the grades in terms of these background characteristics do not explain the differences in the standardized numeracy score across grades.

We find that the standardized numeracy score improves by 119 points between grade 1 and grade 12 – over a full standard deviation gain throughout a child’s entire schooling. Putting this result in context, if we consider what type of trajectory we would expect of a student meeting grade-level expectations, a grade 5 student who was able to correctly answer the relatively easy version of the test (five items that are at grade levels 1–4) correctly would have a score of 238, or more than a 2 standard deviation improvement. In this case, the improvement of 88 points from grades 1–5 is only a third of the improvement in the score that we would expect if all students learned these basic skills. Given that these items reflect content covered in grades 1–5, it is not surprising that most learning takes place during primary school. Between grades 2 and 7, there is an approximate 15-point improvement per grade, or almost a fifth of a standard deviation per grade, compared to an approximate 6-point improvement per grade in grades 8–12.

### 2.2 Did learning change over time?

Because IFLS asked the same questions across survey rounds, it allows us to observe changes in learning between 2000 and 2014. When we apply survey weights, our results for the full sample of respondents between 7 and 18 years old are representative for that population. Table A1.1 shows the balance of the weighted sample between 2000 and 2014. The survey population changed minimally between 2000 and 2014. There were no or very small differences in the gender ratio, age, or distribution of the sample across provinces over time; the main difference was that the population stayed in school longer and was somewhat wealthier.

Fig. 2.4 shows the IRT results for enrolled students and all (in-school and out-of-school) students. The solid lines show the enrolled students’ performance using the standardized numeracy score performance by grade and year. There are negative values in 2014 because we show learning levels relative to the 2000 grade 1 mean, which is standardized to be 0. This does not mean that there was negative learning, but rather that the 2014 grade 1 students performed less well on the test than the 2000 grade 1 students. The striking finding in Fig. 2.4 is that the slopes in 2000 and 2014 are nearly identical, with learning levels slightly higher in 2000. This difference between 2000 and 2014 is statistically significant, as shown in Table A3.1.^10^ Describing this finding another way, a grade 7 student in 2014 performed at the same numeracy level as a grade 4 student in 2000.

The dotted lines in Fig. 2.4 show standardized numeracy score performance for all children, including out-of-school children, by grade (or the grade they would have been in for their age) and year. We include unenrolled children in this analysis to help answer the question of whether the results could be driven by a change in enrollment over time. Enrollment increased between 2000 and 2014, and it increased most for relatively poor children whose mothers completed less than nine years of schooling (authors’ analysis, not shown). Becasue the composition of enrolled students is different in 2014 than in 2000, one might hypothesize that the decline in learning between 2000 and 2014 is at least partly explained by this composition effect.

The enrollment rate for primary school, i.e. grade 1–6, has been nearly universal since before 2000, so the lower numeracy score in 2014 cannot be driven by selection. We can see this in Fig. 2.4 because the dotted and flat lines for both years are nearly identical for grades 1–6. For the secondary schools, as shown in Fig. 1.1, junior secondary school (grades 7–9) enrollment increased by 20 percentage points (from 70 percent to 90 percent) during this time frame; and senior secondary school (grades 10–12) enrollment increased by 24 percentage points, rising from 47 percent in 2000 to 71 percent in 2014. Fig. 2.4 reflects this trend as the 2014 dotted and straight lines are nearly identical through grade 9, whereas the 2000 lines diverge more beyond grade 6.

**Fig. A2.1:**
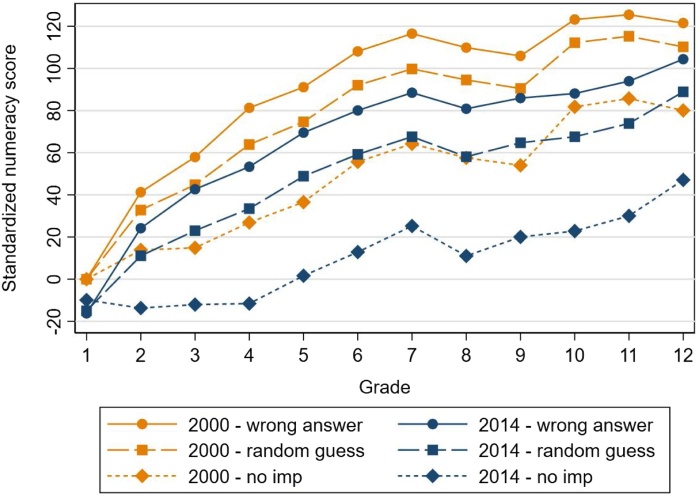
Results when using different imputation methods. **Note**: Results are adjusted for guessing as described in Section 1.4.

**Source**: IFLS 3, 2000 and IFLS 5, 2014 data

Fig. 2.4 shows that learning declined for all children, including enrolled students, between 2000 and 2014, indicating that this difference is not driven by a change in the student composition due to increased enrollment; because there is a consistent difference in learning between the years when we include all children. The difference between 2000 and 2014 is also not driven by our imputation method. Fig. A2.1 shows that we also find a decline in learning if we do not impute or if we consider missing answers as random guessing.

**Fig. 2.5:**
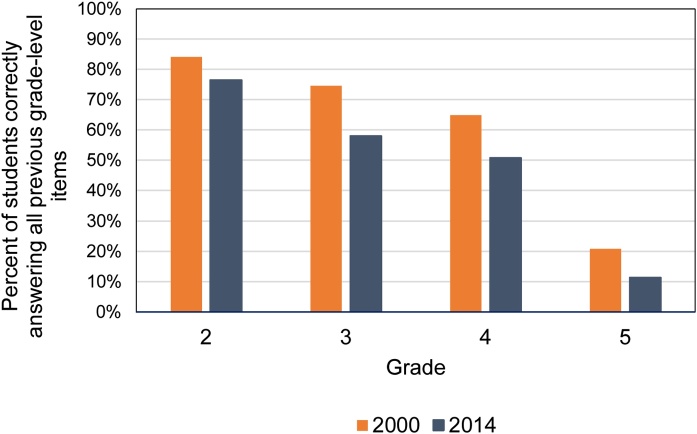
Percent of students who answered items appropriate to their grades in 2000 and 2014. **Note**: Expected grade-level mastery is described in Table 1.1. Figure shows percentage of students enrolled in each grade that correctly answered all items with an expected grade-level mastery below their enrolled grade. Results are not adjusted for guessing as this analysis involves combining items at the respondent level rather than looking at group means that reflect the percent correct of specific items.

**Source**: IFLS 3, 2000 and IFLS 5, 2014

Another way of examining the change in learning over time is to simply look at the share of students answering all relevant grade-level questions correctly. Fig. 2.5 shows that this share is lower for students in every grade in 2014 compared to 2000. For example, we expect that a 4th grader would be able to answer questions for grade 3 and below. In 2000, the share of students who could do this was 65 percent; by 2014, 51 percent of 4th graders answered all grade 1, 2, and 3 level questions correctly. Fig. 2.5 also demonstrates that the decline is not due to a single item since we see this trend across items; and the results are consistent across grade levels.

Above we considered whether learning improved over time for different cohorts of students. Because IFLS is a panel dataset, we can also examine changes in learning among the same respondents in the 2000, 2007, and 2014 surveys, i.e., we can construct a panel learning profile.^11^ In Table 2.1, we look at learning among children who were enrolled in grades 1–5 in either 2000 or 2007, who were also tested seven years later. The “gain” columns show the change in the standardized numeracy score over seven years of schooling for those individuals who were part of the panel, i.e., whom the survey followed over time. For example, those students who were in grade 1 in 2000 gained 62 points between 2000 and 2007.

Consistent with Fig. 2.4, we first find that on average children progressing through grades 1–8 between 2000 and 2007 learned more than the children progressing through the same grades between 2007 and 2014. Learning went down over time. The average gain over seven years for the 2000 cohort was 86 points, whereas this gain was 55 points or half a standard deviation, for the 2007 cohort. The smallest gains were for the older children, i.e., the children in more advanced grades than grades in which much of the material tested would have been taught.

We find that the panel results shown in Table 2.1 are much lower than the cross-section results shown in Fig. 2.3, meaning that this causal learning profile is flatter than the contemporaneous cross-section profile we show in Fig. 2.3. For several cohorts, the change in learning for the cross-section students is double that of the panel students. This indicates that the actual changes in learning were even lower than those shown using the descriptive profile. Because the contemporaneous cross-section profiles are declining, it is logical that the panel profiles demonstrate even lower learning gains.

**Table 2.1:** Change in mean standardized numeracy score between 2000, 2007 and 2014, among panel respondents.

“Baseline” grade	“Endline” grade	Gain in numeracy score from 2000–2007	Gain in numeracy score from 2007–2014
1	8	86.1	54.5
2	9	57.9	47.6
3	10	55.0	29.6
4	11	39.1	18.4
5	12	43.1	15.4

**Note**: Baseline is the year 2000 in column 3 and the year 2007 in column 4, while the endline is the year 2007 in column 3 and the year 2014 in column 4. Results are adjusted for guessing as described in Section 1.4.

### 2.3 Did different subgroups demonstrate different learning profiles?

In addition to looking at learning progress for all children together, we investigate how learning varied across different groups of children, specifically how it varied by gender, wealth quintile, mother’s education level, and province. We also compare differences in learning over time with changes in enrollment between subgroups to explore whether the decline in learning could have been due to changing enrollment. We show these results for enrolled students only, as the primary focus of this analysis is what children learn from the education system. Our findings do not differ significantly when we include out-of-school children. For the analysis in this section, we calculate the subgroup differences by regressing the numeracy score on the subgroup and grade dummies (Table A3.2). Column 1 in Table A3.1 presents the result of a regression of the standardized numeracy score on each of the subgroups and grade dummy variables in 2014 to show the coefficients and significance levels of the differences in that year.

**Fig. 2.6:**
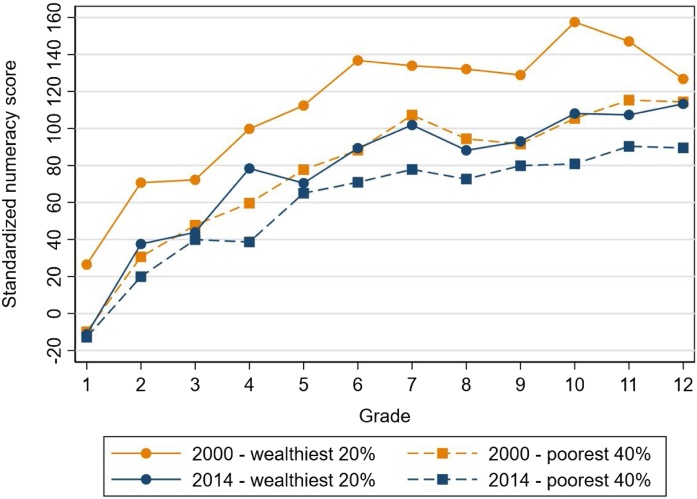
Standardized numeracy score for poorest 40 percent and wealthiest 20 percent in 2000 and 2014. **Notes**: Results are adjusted for guessing as described in Section 1.4.

**Source**: IFLS 3, 2000 and IFLS 5, 2014

In Fig. 2.3, we showed that the standardized numeracy score declined overall between 2000 and 2014. We ask whether this decline was different for different subgroups looking first at the difference between the wealthiest 20 percent and the poorest 40 percent of the in-school population as shown in Fig. 2.6. We determined these wealth categories within each year. The rich-poor gap declined markedly between 2000 and 2014. The mean rich-poor gap per grade was 37 points (about a third of a standard deviation) in 2000 and it went down to 17 points in 2014. As to be expected given the Fig. 2.3 results, learning declined for both groups. This decline was greater for the wealthier group (Table A3.2). The mean 2000–2014 decline per grade was 36 points for the rich and 16 points for the poor (Table A3.2). The results for the rich in 2014 were very similar to the poor in 2000.

We posit that the 2000–2014 decline is a learning effect rather than an enrollment effect due to changes in student composition because the wealthiest 20 percent saw a smaller change in enrollment than the poorest 40 percent, and yet learning still went down for the wealthiest students. Between 2000 and 2014, enrollment rose for the wealthiest 20 percent by 8 percentage points in junior secondary school and 13 percentage points in senior secondary school, while these figures were 27 and 30 percentage points respectively for the poorest 40 percent. If we consider results for all children (not shown), including unenrolled children, we find a similar pattern.

**Fig. 2.7:**
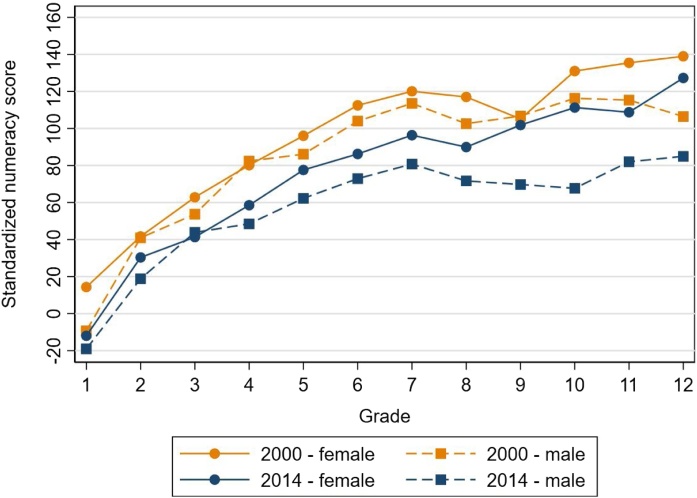
Standardized numeracy score for females and males in 2000 and 2014. **Note**: Results are adjusted for guessing as described in Section 1.4.

**Source**: IFLS 3, 2000 and IFLS 5, 2014

Fig. 2.7 shows similar results by gender. We see that scores declined for both females and males from 2000 to 2014, but that males saw a larger drop and that the male-female gap widened between 2000 and 2014. The average male-female difference in each grade was 10 points in 2000, and this rose to 18 points in 2014 (with females consistently scoring higher). The average decline in scores in each grade from 2000 to 2014 was 20 points for females and 27 points for males (Table A3.2). This was especially high for males after grade 6, where the 2000–2014 difference was 34 points. We do not find a gender difference in attainment over time for primary or junior secondary school. The senior secondary graduation rate difference by gender declined over time; by 2014 the male senior secondary graduation rate was four percentage points higher than that for girls. Thus this gender difference in learning was unlikely due to gender differences in enrollment. Enrollment went up by 14 percentage points for males and 20 percentage points for females in junior secondary school over this timeframe; it rose by 23 percentage points for both genders for senior secondary.

**Fig. 2.8:**
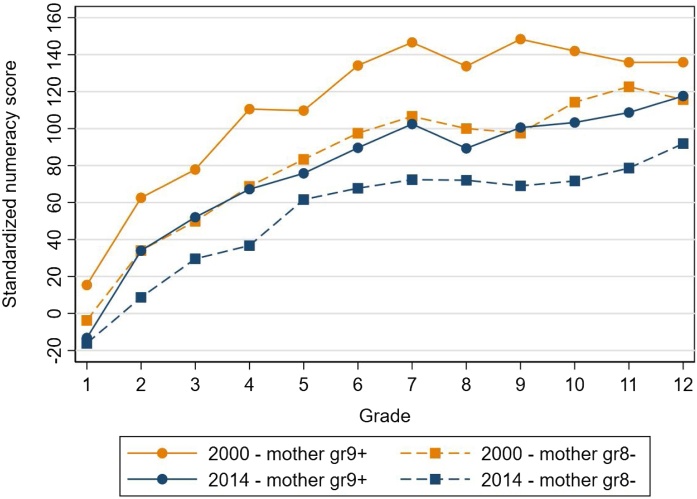
Standardized numeracy score for children whose mothers completed grade 9 and above and whose mothers completed grade 8 or below in 2000 and 2014. **Note**: Results are adjusted for guessing as described in Section 1.4.

**Source**: IFLS 3, 2000 and IFLS 5, 2014

Given that mothers’ education is a strong predictor of educational outcomes (see for example Suryadarma et al., 2006), we also consider how results differ for children whose mothers have different levels of schooling (Fig. 2.8). We use junior secondary school as a cut-off such that we look at differences between children whose mothers completed junior secondary school (grade 9) or above and children whose mothers completed less than junior secondary school (grade 8 or below). Consistent with findings above, we find a decline in learning for both groups over time. The decline is slightly larger for children with mothers with more schooling. Between 2000 and 2014, mean learning within each grade decreased by 36 points for students with mothers who completed at least junior secondary school while it decreased by 28 points for students with mothers with less schooling (Table A3.2). The gap between students with mothers who completed at least junior secondary school and students whose mothers completed less schooling decreased from 31 points in 2000 to 24 points in 2014. Interestingly, in nearly every grade, learning levels among students with mothers with less schooling in 2014 were nearly identical to students with mothers with more schooling in 2000.

As shown in Section 1.1, average years of schooling rose during the 14-year study period, so the share of mothers with a junior secondary degree or above also rose, from 24 percent of students in 2000 to 53 percent in 2014 (Table A1.1). Among children with a mother with a junior secondary degree or above, in 2000, 98 percent of their children were enrolled in junior secondary school (and 93 percent in senior secondary); which confirms that the decline in learning is not due to enrollment changes, at least for this group.

**Fig. 2.9:**
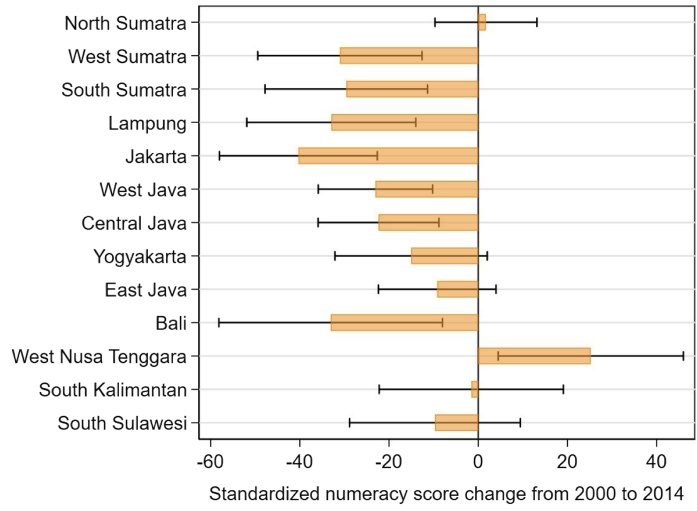
Difference in average standardized numeracy score for students enrolled in grade 1 to 12 from 2000 and 2014, by province. **Note**: Bars present the coefficients and black lines indicate the 95 percent confidence interval of separate regressions for each province of the standardized numeracy score on an indicator for 2014 and grade fixed effects, applying survey weights (β_2_ in Eq. 3). The standard errors are corrected for clustering at the enumeration area level. Results are adjusted for guessing as described in Section 1.4.

**Source**: IFLS 3, 2000 and IFLS 5, 2014

Because educational access and quality varies widely across Indonesia, we might expect a diversity in learning outcomes in different parts of the country. IFLS includes 13 out of 27 provinces and is representative at the province level for the provinces surveyed. Fig. 2.9 shows the change in standardized numeracy test score results for all available provinces. We present the coefficients β_3_ as estimated using Eq. (3) in Section 1.4 for all the 13 provinces that are represented in the IFLS survey. These are the coefficients of the interaction terms between the dummy variable for the 2014 IFLS wave and each of the provinces, showing the difference in the standardized numeracy score between 2000 and 2014 within each province. Not surprisingly, there was a great diversity in mean standardized numeracy scores in 2000. They ranged from 19 points in West Nusa Tenggara to 119 points in West Sumatra, with a mean of 82 points across provinces. We find that scores declined in all but three provinces. Only one province, West Nusa Tenggara, which had the lowest baseline score, saw a positive and significant difference; declines were significant for 7 out of 13 provinces. In Jakarta, which started with an average score of 109 in 2000, the average score declined up to 40 points, or a bit over a third of a standard deviation. Again, we find a larger decline for groups with initially higher scores. The provinces with a significant decline in the numeracy score had an average standardized numeracy score in 2000 of 92; the provinces with no change had an average initial score of 76.

## 3 Discussion and conclusion

Between 2000 and 2014, Indonesia witnessed major progress in junior and senior secondary enrollment, as shown in Fig. 1.1: a growth of 20 percentage points in junior secondary schools and 24 percentage points in senior secondary schools. Average years of schooling completed among 18 to 24-year-olds went up by 1.4 years over this 14-year time frame. We find that despite this progress, learning levels remained low. For example, looking at the simplest question in our study, a grade 1 question, 49−23, 65 percent of students in grade 3 in 2014 were able to answer it correctly. No 5th graders answered a more difficult question, 1/3−1/6, a grade 4 question, correctly. We find that the disparity between subgroups in terms of ability grew as the questions grew in difficulty.

In a study that tested children in grades 1–9 at two points in time, in 2011 and 2012, Afkar et al. (2018) also find similarly low levels of learning in Indonesia. Just 57 percent of children could correctly answer a one-digit multiplication question by the end of grade 3; 50 percent could order four-digit numbers from big to small by the end of grade 2; and 60 percent could recognize two-digit numbers by the end of grade 2. PISA and TIMMS results also reinforce this finding of similarly low learning levels (OECD, 2019; Mullis et al., 2016).

We further show that learning *declined* over 14 years. This decline amounted to approximately one-fourth of a standard deviation based on a scale normalized to grade 1 learning levels in 2000. This decline was the equivalent of nearly three grades of learning; the average grade 7 student in 2014 demonstrated the same numeracy mastery as the average grade 4 student in 2000. Comparing these results to international assessments, Indonesia’s TIMSS scores declined for grade 8 mathematics between 2003 and 2011 (Luschei, 2017). In PISA, mathematics scores over a similar timeframe (2003–2018) improved by just a few points on average over the six PISA tests that Indonesia participated in (OECD, 2019).

A critical outstanding question is why learning declined. There are several reasons to suggest it was not due to the changes in enrollment. First, we see a decline in learning at the primary level while primary school enrollment was basically universal by 1988. If there was a compositional effect at higher grades, we would expect to see differences in the decline in these grades compared to primary – which we don’t.

Second, looking at the entire population (in- and out-of-school children) across all ages, we still see a decline, as shown in Fig. 2.4; so there wasn’t a selection effect. The decline for the children in school is greater in magnitude than the improvement in learning for the children who entered school and wouldn’t have otherwise. Taking all 18-year-old respondents in 2014, using 2014 enrollment levels but the 2000 learning profile, we would expect them to have an average standardized numeracy score of 100; but instead they have an average score of 73 due to the declining learning profile. It is possible that learning for in-school children declined due to increased enrollment because more students stressed the system (and thus lowered quality for all) or due to peer effects from new learners who were not in school in 2000. However, our finding that learning also declined at the primary level where enrolment did not change between 2000 and 2014 makes the case against system stress or negative peer effects, unless those challenges were unique to grades 7–12.

Third, learning declined for nearly all subgroups, even those that had high levels of enrollment in 2000. For example, learning actually declined more for the wealthiest 20 percent than for the poorest 40 percent and for children with mothers with more education than for children with less education, despite the fact that enrollment changed less for these subgroups. Between 2000 and 2014, enrollment rose for the wealthiest 20 percent by 8 percentage points in junior secondary school and 13 percentage points in senior secondary school. Ninety-eight percent of children with a mother with a junior secondary degree (93 percent for senior secondary) were already enrolled in junior secondary in 2000 and enrollment for this group did not change much by 2014.

The learning decline is especially surprising given all the education system upgrades that took place over this timeframe. These include nationwide decentralization in 2001 to allow districts more flexibility with introducing innovative education policies and adjusting policy to reflect local context; the 2002 amendment to the Constitution that required 20 percent of the budget be devoted to education expenditures—resulting in a threefold increase in real education budget; and the 2005 teacher certification policy as a way to improve teacher quality. The increased budget allowed for a decline in the student teacher ratio during this period and one aspect of teacher quality, the share of teachers with bachelor’s degree, rose from 37 to 90 percent (World Bank, 2018a).

However, many of these policies were not directly targeted at learning or specifically at improving foundational skills like the numeracy questions analyzed in this paper. Given the mixed evidence of the impact of spending on learning, it is not guaranteed that the 2002 budget requirements on education spending would have had an impact on learning (Vegas and Coffin, 2015; World Bank, 2018b). Indeed, a study examining the impacts of the teacher compensation component of the teacher certification law of 2005 showed that it had no impact on learning (de Ree et al., 2018). Districts could use greater education policy autonomy to achieve goals that are not necessarily aligned with improving student learning, such as satisfying certain constituent demands for job opportunities within the school system.

What then could have caused the learning decline? In the absence of a causal study, we only have several conjectures. First, as mentioned in Section 1.1, children’s exposure to math changed over this timeframe. The 1994 curriculum mandated 10 hours a week of math instruction for grades 1–3 and eight hours a week for grades 4–6. In 2004, the curriculum was to be taught “thematically” for grades 1 and 3, and instruction time went down to five hours per week for grades 4–6. Of course it is possible that thematic teaching was a more efficient and holistic way of learning; but cutting math instruction time in half could potentially have an effect on learning.

Second, related to dosage or exposure to material, grade repetition went down by 38 percent (from 17 percent in 2000 to 11 percent in 2014), indicating that perhaps students who might have needed more support by repeating a grade would have been able to in 2000 but not in 2014 (authors’ analysis with IFLS, not shown). By 2014, fewer children were behind grade level and more children were either at the appropriate grade level for age or ahead (meaning young for their grade) compared to 2000. For the richest 20 percent, the percent of students repeating a grade dropped from 14 to 6 percent, and for the poorest 20 percent, this only declined from 19 to 17 percent. Thus it is possible the decline in grade repetition for the rich contributed to the learning decline, although we would not expect this to have a very large overall effect given that the decline across all groups was 6 percentage points.

Third, class grades became less important which could have affected student incentives to learn. Prior to 2003, a student graduated from 6th, 9th, or 12th grade based on yearly grades and national exam results. After 2003, grades were less important as graduation was determined by a combination of teacher discretion and national exam results. During this timeframe, districts took over responsibility for the grade 6 leaving exam, so the content varied by district. Thus the weight of exams in graduation could have affected incentives for learning during the 2000–2014 timeframe.

Consistent with many studies outside of Indonesia, importantly the World Bank’s World Development Report 2018 (World Bank, 2018b), this study makes it clear that rising enrollment does not necessarily translate to improved test performance. Indonesia took costly measures to address education challenges over the 2000–2014 timeframe and yet not only did learning not improve but it declined. This study shows that policy should more carefully explore and target the major barriers to learning, which in Indonesia appear not to be financing, teacher qualifications, or teacher-student ratios; they could be the duration of exposure to mathematics or incentives to learn, but more study is needed to uncover the primary barriers to improving learning. Moreover, this study emphasizes the importance of comparable, low stakes exams that ask similar questions over time for monitoring purposes. We hope that this study will encourage more government-supported outcomes monitoring, a key starting point to any strategy that seeks to transform education systems and prioritize learning.

## Author statement

Amanda Beatty: conceptualization, methodology, writing - original draft, writing - review & editing, visualization, supervision, project administration, funding acquisition

Emilie Berkhout: conceptualization, methodology, software, validation, formal analysis, investigation, data curation, writing - original draft, writing - review & editing, visualization

Luhur Bima: conceptualization, formal analysis, writing - original draft

Menno Pradhan: conceptualization, methodology, writing - original draft, writing - review & editing, funding acquisition, supervision

Daniel Suryadarma: conceptualization, methodology, formal analysis, writing - review & editing, funding acquisition

## Funding

This research was funded under the RISE Programme by the United Kingdom’s Foreign, Commonwealth & Development Office (FCDO), the Australian Government’s Department of Foreign Affairs and Trade (DFAT), and the 10.13039/100000865Bill and Melinda Gates Foundation.

## Figures and Tables

**Table A1.1:** Balance between the IFLS sample in 2000 and 2014.

	(1)	(2)	(3)
	2000	2014	Difference
Age in Years	12.41	12.23	−0.18***
	(3.49)	(3.28)	(0.05)
Fraction Male	0.52	0.52	−0.00
	(0.50)	(0.50)	(0.01)
Completed Years of Schooling	4.99	5.50	0.51***
	(3.31)	(3.22)	(0.07)
Fraction of Mothers that Completed at least Junior Secondary School	0.24	0.53	0.29***
	(0.43)	(0.50)	(0.01)
Standardized Asset Index	0.06	0.23	0.17***
	(0.96)	(0.83)	(0.03)
Fraction Living in […]			
*North Sumatra*	0.06	0.07	0.01***
	(0.23)	(0.25)	(0.00)
*West Sumatra*	0.04	0.04	−0.00
	(0.19)	(0.19)	(0.00)
*South Sumatra*	0.04	0.04	−0.00
	(0.20)	(0.20)	(0.00)
*Lampung*	0.04	0.03	−0.01
	(0.20)	(0.18)	(0.00)
*Jakarta*	0.05	0.05	0.00
	(0.21)	(0.22)	(0.00)
*West Java*	0.28	0.24	−0.04***
	(0.45)	(0.43)	(0.01)
*Central Java*	0.15	0.17	0.02**
	(0.36)	(0.37)	(0.01)
*Yogyakarta*	0.05	0.04	−0.01*
	(0.22)	(0.21)	(0.00)
*East Java*	0.19	0.19	0.00
	(0.40)	(0.40)	(0.01)
*Bali*	0.02	0.02	0.00
	(0.12)	(0.13)	(0.00)
*West Nusa Tenggara*	0.03	0.03	0.00*
	(0.16)	(0.17)	(0.00)
*South Kalimantan*	0.03	0.03	0.00
	(0.17)	(0.17)	(0.00)
*South Sulawesi*	0.04	0.04	0.00
	(0.19)	(0.20)	(0.00)

Note: Table includes all respondents between 7 and 18 years old, and respondents older than 18 years that are still enrolled in senior secondary school. Values are weighted using the sampling weights. Standard errors in parentheses and corrected for clustering at the EA level.

* *p* < .10, ** *p* < .05, *** *p* < .01.

**Table A2.1:** Fraction missing by item and age.

Item / Age	7	8	9	10	11	12	13	14	15	16	17	18
G1: 49-23	0.19	0.09	0.05	0.04	0.03	0.02	0.02	0.02	0.01	0.01	0.02	0.02
G2:267+112-189	0.26	0.13	0.07	0.05	0.04	0.03	0.03	0.04	0.02	0.02	0.03	0.02
G3: (8+9)*3	0.33	0.17	0.10	0.07	0.05	0.04	0.04	0.04	0.03	0.02	0.04	0.03
G3: (412+213)/(243-118)									0.07	0.07	0.08	0.08
G4: 56/84	0.45	0.34	0.21	0.16	0.10	0.08	0.06	0.06	0.05	0.04	0.07	0.05
G4: 1/3-1/6	0.45	0.33	0.19	0.14	0.10	0.07	0.05	0.05	0.04	0.03	0.05	0.04
G4: 0.76-0.4-0.23									0.07	0.07	0.09	0.09
G5: Percent 1									0.08	0.08	0.09	0.09
G5: Percent 2									0.08	0.07	0.08	0.08

**Table A3.1:** Subgroup differences in standardized numeracy score in 2014 and the difference in the standardized numeracy score between 2000 and 2014.

	(1)	(2)	(3)
	Subgroup Comparison 2014	Difference over Time	Difference over Time with Controls
	Standardized Numeracy Score		
Male	−18.721***		−14.373***
	(2.294)		(1.602)
Poorest 40 %			
Middle 40 %	5.003*		6.942***
	(2.610)		(2.237)
Wealthiest 20 %	7.766***		16.067***
	(2.964)		(2.690)
Mother completed at least junior secondary school	21.269***		22.624***
	(2.705)		(2.147)
Year 2014		−23.698***	−29.461***
		(2.156)	(2.289)
Constant	−19.917***	2.746	−6.101
	(7.658)	(4.839)	(6.364)
Province Fixed Effects	Yes	No	Yes
Grade Fixed Effects	Yes	Yes	Yes
Years Included	2014	2000 and 2014	2000 and 2014
Observations	9133	16,873	15,993

Standard errors in parentheses and corrected for clustering at the EA level.

* *p* < .10, ** *p* < .05, *** *p* < .01.

**Table A3.2:** Subgroup differences in the change in the standardized numeracy score between 2000 and 2014.

	(1)	(2)	(3)	(4)	(5)	(6)	(7)
	Standardized Numeracy Score by Gender		Standardized Numeracy Score by Wealth			Standardized Numeracy Score by Mother’s Education	
	Female	Male	Poorest 40 %	Middle 40 %	Wealthiest 20 %	Completed less than JSS	Completed at least JSS
Year 2014	−19.968***	−27.108***	−16.212***	−21.472***	−35.772***	−28.023***	−36.010***
	(2.716)	(2.676)	(3.317)	(3.159)	(3.879)	(2.828)	(3.224)
Constant	11.971*	−2.971	−5.311	−0.265	25.596***	0.070	19.813***
	(6.288)	(6.072)	(7.254)	(6.145)	(8.687)	(5.787)	(6.541)
Grade Fixed Effects	Yes	Yes	Yes	Yes	Yes	Yes	Yes
Observations	8258	8615	6707	6433	3662	8918	7421

Models include enrolled students in grade 1–12 in 2000 or 2014. Standard errors in parentheses and corrected for clustering at the EA level. JSS stands for junior secondary school.

* *p* < .10, ** *p* < .05, *** *p* < .01.

## Appendix A

### A1 Balance Between the 2000 and 2014 Sample

The table below shows the difference in characteristics between the sample included in the 2000 IFLS sample and the 2014 IFLS sample. Applying the sampling weights, the samples are representative for the population between 7 and 18 years old in the 13 provinces in each of these years. Since the population can change over time, we do not expect the samples to be the same. The sample in 2014 is slightly younger than the one in 2000 (0.2 years), they completed half a year of schooling more, and 30 percentage points more mothers completed at least junior secondary school. The population also improved their wealth with 0.2 standard deviation. The gender ratio and the distribution of the sample across the provinces remained virtually the same. Note that we standardize the asset index and determine the wealth quantiles separately in each year at the household level. Since there can be multiple respondents in one household, the fraction can be slightly different at the individual level.

#### A2 Different Imputation Methods as Robustness Checks

We conduct several tests to assess the robustness of our findings to different imputation specifications. Our primary results are presented using imputations of wrong answers for (partial) missing cases, meaning that we assume that a student did not know the answer to the question if he or she left the field blank. We think that the latter is likely to be the case, because there are more missing values amongst younger kids and more difficult items (see Table A2.1). In Fig. A2.1, we present our primary approach (impute with wrong answers or 0), the standardized numeracy score when not imputing missing values and the standardized numeracy score if we would impute with random guessing. Children that did not know the answer to the question could make a guess instead of leaving the field blank. When a question has 4 answer options, we impute 25 percent of the missing values randomly with a correct answer.

Overall, we find that results from our primary approach are similar to results without conducting any imputation and to results when imputing missing values with random guessing. The other imputation methods result in a somewhat flatter learning profile, but in all cases most learning takes place between grade 1 and 6 and the learning profile declines between 2000 and 2014.

The learning profile from our primary imputation approach is steeper, because ignoring missing values and imputation with random guessing inflate scores of children in lower grades. We standardize such that the grade 1 mean is 0 and the grade 1 standard deviation is 100. There are more missing answers for students in lower grades, especially for grade 1 students, so if we assume that all missing values are wrong answers, it makes sense that we find more learning over grades than with the other methods.

##### A3 Regression Analysis of Subgroup Differences and Differences over Time

As part of our subgroup analysis, we also use regression analysis to examine what factors might explain learning differences among children in the same grade. Table A3.1 shows that the differences between the subgroups and over time that we described in section 2.3 are significant.

We test the significance of the sub-group differences and differences over time using three regressions.

First, we test the significance of the differences between the subgroups in the 2014 IFLS wave by regressing the standardized numeracy score on subgroup indicators, controlling for grade in which the student is enrolled and weighting the observations using the sampling weights, as shown in Eq. 1 for individual *i* from province *p* and grade *g*,

(1) $$Y_{i,p,g}=\beta_{1}+\beta_{2}{MALE}_{i,p,g}+\beta_{3}{SES}_{i,p,g}+\beta_{4}{MOTH\_EDUC}_{i,p,g}+\varphi_{p}+\gamma_{g}+\varepsilon_{i,p,g}$$

In which Y is the standardized numeracy score that follows from IRT. MALE, SES and MOTH_EDUC are dummy variables indicating the subgroups, φ_p_ are province fixed effects, γ_g_ are grade fixed effects and ε is an error term. We estimate the model using Ordinary Least Squares (OLS) and the standard errors are corrected for clustering at the enumeration area level.

Second, we test the significance of the difference in the standardized numeracy score over time by including the 2000 IFLS wave and by adding a dummy for the 2014 IFLS wave to Eq. (1). This way, we test whether the difference over time is significant while controlling for background characteristics and grade as shown in Eq. 2 for individual *i* in IFLS wave *w* and grade *g*,

(2) $$Y_{i,p,w,g}=\beta_{1}+\beta_{2}{MALE}_{i,p,w,g}+\beta_{3}{SES}_{i,p,w,g}+\beta_{4}{MOTH\_EDUC}_{i,p,w,g}+\beta_{5}W_{i,p,g}+\varphi_{p}+\gamma_{g}+\varepsilon_{i,p,w,g}$$

In which W is a dummy variable for the 2014 IFLS wave.

Table A3.1 shows the results of the regression analysis. All subgroup differences in the standardized numeracy score are statistically significant in the 2014 sample, except for the difference between the 40 % poorest and 40 % middle SES students. The differences by gender and mother’s education are the largest, where girls and students with mother’s that completed at least junior secondary school scored about a fifth of a standard deviation higher on the numeracy test. The decline in the standardized numeracy score of enrolled students between 2000 and 2014 is 29 points and statistically significant, even when controlling for the background characteristics of students.

Third, we test the significance of the difference in the standardized numeracy score over time for each of the subgroups by estimating the following equation for each of the subgroups separately,

(3) $$Y_{i,w,g}=\beta_{1}+\beta_{2}W_{i,g}+\gamma_{g}+\varepsilon_{i,w,g}$$

For student *i* from IFLS wave *w* in grade *g*. Again W is a dummy variable for the 2014 IFLS wave and we include grade fixed effects γ_g._ Note that the grade fixed effects are allowed to differ between the subgroups. Also note that we estimate the same model for each of the provinces, for which we show the results in Fig. 2.8 in section 2.3.

The results in Table A3.2 show that the standardized numeracy score significantly declined for all subgroups. It declined more for boys, for wealthier students and for students whose mothers completed at least junior secondary school. With almost two fifths of a standard deviation, the standardized numeracy score declined most for the wealthiest 20 % and for students whose mothers completed at least junior secondary school.

## Footnotes

1 All analysis in this paper focuses on all school types combined. This includes secular public schools, religious public schools, and secular and religious private schools.

2 The discrepancy between the IFLS and the national statistics likely reflects the fact that the IFLS is representative of 83 percent of the population and the omitted 17 percent represents mainly very remote areas.

3 The IFLS over-sampled rural enumeration areas and enumeration areas in smaller provinces to facilitate urban-rural and Javanese-non-Javanese comparisons. We use sampling weights to correct for this.

4 Note that there are no anchor groups in the 2000 survey. The numeracy score is based on the anchor respondents in 2007 and 2014. Technically, we assume that the relative difficulty levels and discrimination power of the items remained the same over time and is the same across the country.

5 We use the openIRT Stata program developed by Tristan Zajonc. Maximum likelihood estimates of latent ability are similar and available upon request.

6 We check the validity of the score with factor and infit and outfit analysis, and we examine the reliability using Cronbach’s alpha and the IRT discrimination coefficients. In addition, we run tests on the IRT assumptions of unidimensionality, no differential item functioning, and conditional local independence.

7 Standardizing using the grade 1 mean and standard deviation could result in unrealistically large difference in learning across grades, because we might expect the grade 1 standard deviation to be relatively small as the test is actually too difficult for these students. However, our results look similar when we use the grade 5 standard deviation for the standardization. For ease of interpretation (improvements relative to grade 1), we use the standardization using the grade 1 mean and standard deviation.

8 The included assets are a house, land, other buildings, poultry, livestock or fish pond, vehicles (cars, boats, bicycles, motorbikes), household appliances (radio, television, fridge, etc.), savings or certificate of deposit or stocks, credits (money owed to the household), jewellery, and household furniture and utensils.

9 These are North Sumatra, West Sumatra, South Sumatra, Lampung, Jakarta, West Java, Central Java, Yogyakarta, East Java, Bali, West Nusa Tenggara, South Kalimantan and South Sulawesi.

10 As a robustness check, we checked whether this result is driven by differential item functioning between the years. This is not the case. Results are available upon request.

11 We do not consider the 2007 survey in any other analysis in this paper since 2007 is more of a midterm result and does not add to existing information about the learning decline other than to confirm it.
